# “It Makes Me Feel like I Can Make a Difference”: A Qualitative Exploration of Peer Mentoring with Black and Hispanic High School Students

**DOI:** 10.3390/youth3020034

**Published:** 2023-04-03

**Authors:** Ijeoma Opara, Isha W. Metzger, Sandy Dawoud, Kimberly Pierre, Maame Araba Assan, Pauline Garcia-Reid, Robert J. Reid

**Affiliations:** 1 Department Social & Behavioral Sciences, Yale School of Public Health, New Haven, CT 06510, USA; 2 Department of Psychology, Georgia State University, Atlanta, GA 30302, USA; 3 Department of Family Science & Human Development, Montclair State University, Montclair, NJ 07043, USA; 4 Irvington Department of Health and Senior Services, Irvington, NJ 07111, USA; 5 Rutgers Cancer Institute of New Jersey, Rutgers, The State University, Newark, NJ 07103, USA

**Keywords:** mentors, resilience, strengths, youth of color

## Abstract

Peer mentoring programs have proven to be extremely successful for high school students. Yet, most educational research studies rarely seek to understand the perspectives of peer mentors and the impact peer mentoring can have on their development. Even more limited is the research highlighting the experiences of Black and Hispanic peer mentors who reside in urban communities. This qualitative study examines (*n* = 14) Black and Hispanic high school peer mentors’ roles in providing support to their mentees and their perceived benefit of being a mentor. All peer mentors in the study attended high school in an urban, under resourced community in New Jersey. Analysis revealed three major themes: (1) leadership abilities; (2) witnessing their strengths through motivating others; and (3) Family influences on their mentoring style. We discuss the implications of our findings on future research and educational programming utilizing peer mentors to benefit urban youth of color.

## 1. Introduction

Education research that focuses on urban communities and school districts in the U.S. tends to view urban youth of color through a deficit lens [1]. This deficit perspective often ignores the resiliency skills of youth of color in urban communities and their ability to thrive in under-resourced environments [2,3]. Unfortunately, many youth living in urban and under-resourced areas face systemic factors that are beyond their control that can contribute to lower educational attainment such as lack of quality neighborhood resources [4,5] and exposure to community violence and trauma [6,7]. Despite these factors, education research has not fully explored effective strategies, such as peer mentoring, that can contribute to resiliency among urban students of color. Mentoring involves an experienced individual (mentor) supporting and guiding another individual who requires guidance (mentee) in any given area. While there can be power dynamics and hierarchical dynamics at play, peer mentoring provides an equitable solution to this issue [8]. Structured peer mentoring in high schools can prove to be more relatable, less controlling, and help students of color to form relationships that can improve their lives. Peer mentoring, is rooted within the belief that it can be beneficial for youth to receive support from their fellow peers who are similar in age, rather than from adults. Peer mentoring programs, based on the literature, have shown to be effective on youth development, particularly for urban youth mentees [9–11].

Over the past few decades, there has been a growing interest in the field of peer mentoring in high schools in the U.S. [12,13]. Peer mentors in high schools may serve as positive role models by offering friendship, constructive review, reinforcement, and general counsel depending on the nature of the relationship and instructional approach [14,15]. High school peer mentoring programs have numerous benefits to the mentee including promoting self-esteem, building networking opportunities, adjusting to high school, addressing mental health, and making informed future career choices [16–20]. Notably, peer mentorship programs allow opportunities for mentors and mentees to build meaningful relationships and establish trust [21]. The group dynamics established within peer mentorship program enable the students to alternate between seeking counsel, supporting each other, and building their self-confidence [16,22,23] Therefore, a well-structured and purposeful relationship may provide mentees with social and emotional support, enhancing their confidence and motivation in academic and life goals.

Involving and engaging urban youth in their communities through peer mentoring allows them to develop leadership skills and improve their academic ability [24]. While the benefits of peer mentoring on mentees is well-established in the literature, there is limited evidence to show the benefit of mentoring for mentors themselves. Examining their perspectives can lead to support in developing structured peer mentoring programs in order to maximize benefits for mentees and mentors within high school settings. High school students’ proactive involvement in mentorship programs that serve as mentors can contribute to the construction of a better society through active citizenship [25]. In addition, peer mentoring programs provide high school students who are mentors, with an opportunity to learn and perceive volunteering and community development as an essential part of their educational experience, personal life, and future career choice [26]. This development and experience can contribute to their social and interpersonal skills, developing a citizenship culture and a sense of responsibility among youth.

The few studies that have examined the impact of peer mentoring on mentors showed that mentoring programs provided students who were mentors with leadership skills and a sense of responsibility [25,27]. In another study conducted among Indigenous high school youth, the researchers found as a major theme in their study that being a peer mentor provided benefit to them to increase their own self-esteem and self-worth due to being a mentor [28]. While there is extensive research outlining the benefits of developing mentoring relationships from a mentee perspective, there is limited research exploring whether peer mentors in urban, under resourced communities have similar positive benefits as peer mentees. While research on urban youth is often portrayed through a deficit lens, it is essential for researchers to highlight the strengths of youth that are working in under resourced environments and schools and are still able to thrive [4,5,7].

## 2. Purpose of Study

Despite evidence that peer mentoring programs provide benefit to student mentees, few studies have aimed to elicit an in-depth exploration of the experience of being a peer mentor in an urban environment. Providing peer mentoring is a lived experience for the youth involved and understanding their perspectives is another way of recognizing and highlighting their strengths. This study, therefore, aims to explore the perceived benefit of peer mentors in an urban high school.

## 3. Methods

### 3.1. Community Context

This study was conducted in one of the most diverse cities in New Jersey, with 61.4% of residents identifying as Hispanic, 25.7% identifying as African American/Black, followed by 27.2% identifying as White [29]. Approximately 43.3% of residents living in this city were foreign born [30], and approximately 25.2% live below the poverty line, with a median household income of USD 33,000 yearly, compared to 10.4% living below poverty line and a median income of USD 71,180 for the entire state [29]. The city is one of the poorest cities in New Jersey, with a median income that is among the lowest in the state [29]. The city’s child poverty rate is 41%, which is higher than New Jersey’s rate of 16% of children living in poverty [31].

### 3.2. Sampling

The lead facilitator (I.O.) worked with the established peer mentoring group at the local charter high school in New Jersey on various activities including a Photovoice project [32], which was intended to display issues pertaining to drug use and sexual health that the students wanted to tackle. Through this engagement, the peer mentors and facilitator established a relationship where the peer mentors wanted to make a difference in their community through art and to continue working closely with the research team. Because the goal of descriptive phenomenology is to describe the common features of participants’ experiences, participants shared a restricted range of demographic characteristics. The participants were all in the same grade (12th grade) and all participated as peer mentors. The sample includes (*n* = 14) peer mentors who all attended the same high school (see demographics in Table 1). All peer mentors at the selected school consented to being a part of the study, with the exception of one peer mentor who did not consent and was not included in the study. The study was approved by Montclair State University Institutional Review Board. Participants had to provide signed written consent, and parental consent was attained for participants who were under the age of 18 years old at the time of the study. The facilitator (I.O.) formed research questions around the goals and formation of the peer mentoring group with the intent of highlighting the strengths and any potential areas of improvement as a contribution to the literature on peer mentoring programs in urban communities. The peer mentors were all high school seniors who were graduating from high school during the year the study was conducted, 2019. All participants were given aliases to protect their identities.

The first author and one masters-level and one doctoral-level research assistant conducted the interviews. In accord with the descriptive phenomenology method, the researchers’ knowledge of the literature and their experiences with the phenomena of interest were set aside in memos and notes. Participants were compensated with a USD 5 gift card for each interview. Interviews occurred during school hours during the peer mentor’s intervention period in the library or in a guidance counselor’s office with permission from guidance counselors. All interviews were private and in closed rooms in the school. Semi-structured interviews were utilized to encourage informants to speak freely and to minimize the elicitation of the interviewers’ preconceived ideas. The first question was broad, asking respondents to describe their story of how they became a peer mentor. Asking such a broad question allowed the participants to direct the content of the interview. The interviews were instructed to focus on eliciting further description by probing. Probes included asking them to “tell me more” about something that was said or what they did when a particular event occurred or to “describe” what something meant. Participants were encouraged to focus thoughtfully on their experience and journey to becoming a peer mentor. Interviews lasted approximately 45 min each.

### 3.3. Researcher Positionality

As part of a larger federal substance abuse and HIV prevention grant initiative in the community, we have engaged with many participants as educators, mentors, and/or advocates for their needs in substance-abuse prevention. Moreover, we have working relationships with youth-serving community organizations. In establishing credibility, we needed to obtain trust and develop rapport with the communities we serve through prolonged engagement [33]. Through prolonged engagement with the high school, we were able to build a rapport with the guidance counselors, staff, and peer mentors, which enabled researchers to establish trustworthiness strategies. Prolonged engagement, in conjunction with persistent observation (intense focus on the aspects of setting and phenomenon), allowed researchers to spend considerable time in the field to thoroughly understand students’ perspectives and to offset the researchers’ own bias [34]. As Lincoln (1989) and Guba (1985) note, “if prolonged engagement provides scope, persistent observation provides depth”. We acknowledge that we come from privileged social locations, although some of us identify with intersectional perspectives of race, ethnicity, gender, and upbringing. We acknowledge that we are not experts, nor insiders, into the daily issues and lived reality of the urban youth who were a part of the study.

### 3.4. Data Analysis

The methodology and analysis by the interviewers and authors of this work were informed by a strengths-based, empowerment-theoretical framework [35]. Interviews were recorded and transcribed verbatim by doctoral- and masters-level research assistants. Transcripts were listened to and verified by the first author in order to check for accuracy. Analysis of interview data involved four coders. Analysis was informed using grounded theory methodology to analyze the 14 interviews [36]. The first step in grounded theory methodologies is to conduct open coding. As such, the coders (IO, SD, KP, and MAA) first identified codes through a round of open coding by reading each of the transcript’s multiple times. The four read and re-read the transcripts, coded the transcript, and met weekly to discuss the findings. Analysis proceeded inductively through the identification of recurring themes and patterns in transcripts, field notes, and analytic memos. Meaningful analytical units were then developed by using a coding scheme that was informed by dominant themes in the data. In the next round of coding [37,38], research assistants identified subcodes within the larger aforementioned categories. These topics were then divided into several subtopics based on recurring themes within the larger topics, allowing for more in-depth analysis and complex understanding and interpretation of each particular theme. Each theme and subtheme assigned a code, and the codes were compiled in a codebook. The authors then clarified the codes’ definitions and ensured that all codes fit into a structure with meaningful and salient inter-relations and distinctions among them. Subcodes were identified both inductively and deductively as a way to examine if emerging themes from the participants had any relationship with aspects of previous literature. Subsequently, the relationship between concepts and categories was analyzed. Quality checks were undertaken to ensure high inter- and intra-coder reliability among coders. Given these initial findings, the final phase of coding [37,39] involved examining the interviews in search of the influence of the major themes. After considerable discussion of apparent themes and associations between codes, the research team wrote memos for all participants’ interviews on the association between the major themes that arose involving leadership abilities, motivating their peers, and family dynamics. Three major themes were identified and are described in the sections below. The resulting data were utilized to examine the specific research questions guiding the proposed study.

While the authors aimed to understand the participants through a strengths-based lens and highlight the voices of urban youth of color, the authors acknowledge that power imbalances between participants and researchers and the perspectives with which their experiences are viewed may certainly shape the reported findings from these interviews. Thus, the authors recognized power differentials, their own ethnic and socioeconomic backgrounds, and other differences from the participants. Through reflexivity and member checking, the authors sought to ensure that the participants stories were accurately being told. Member-checking occurred after all 14 interviews were completed and analysis was conducted. The first author visited the school and all students that completed the interviews were debriefed about the findings as a group to confirm whether findings accurately captured the researchers’ view. Memos and notes were taken by the facilitator and were used as preliminary analyses to report back to the students.

## 4. Findings

The findings of this study summarize the peer mentor’s experiences in providing mentoring to their younger high school counterparts. Three inter-related themes were identified from the data supporting the key theme of resilience and empowerment: (1) leadership ability; (2) witnessing their strengths through motivating others; and (3) importance of family influence. The participants described their experiences of being a peer mentor and how it changed their perspective of themselves and their trajectories. As the peer mentors told their stories, they all consistently mentioned that seeing their mentees look up to them was motivation for continuing the mentorship path. Of note, all of the peer mentors were motivated to help others, and they began to recognize the value of overcoming their own insecurities and fears to inspire their mentees.

### 4.1. Leadership Ability

All 14 peer mentors agreed that taking on the role of a peer mentor gave them the opportunity to strengthen their leadership skills and to be seen as leaders in their school community. Participants mentioned that they were initially chosen to be a peer mentor by their guidance counselor when they were sophomores in high school and tasked to mentor eighth graders. At the time of the study, all peer mentors had been mentors for at least 2 years. The criteria for selecting peer mentors was not disclosed to students. Some participants mentioned that they were already high-achieving students, while others mentioned that they were surprised to have been selected as a peer mentor. Vicky, for example, mentioned her story to becoming a peer mentor and how the peer mentoring program had a mixture of students who were considered leaders in their group and not necessarily those who made good grades:

“Because I see a lot of peer mentors who are not too successful academically, but are leaders amongst their group; because either they’re more outgoing or they’re able to speak to more people. So for me, when they chose me to be a peer a mentor, I was shocked because I just usually just stay by myself. I do get good grades, but it took me time to get comfortable talking to me. I guess they saw something in me”.

In contrast, Kevin knew he was a leader amongst his peers before becoming a peer mentor. Kevin displayed a lot of confidence and acknowledged that he has always had a “gift” of being outspoken and secure in himself since he was a young child. Kevin attributed these qualities to why he was selected to be a peer mentor:

“When I was asked to be a peer mentor, I wasn’t surprised. I have good grades. I’m a nice person … I feel like people in this school look up to me a lot. When people have problems and stuff and they come to me; Like I’m that neutral level-headed person that everyone loves”.

Tamara, another peer mentor, also mentioned: “Before this program I didn’t think I had it in me to be a leader and get these kids talking. But after, peer mentoring gives me that leadership role to advise these kids in a way”.

In discussing their experiences, all 14 of the peer mentors mentioned that being a peer mentor contributed to their understanding of what it means to be a leader, including Veronica, who attributed her behavior to not wanting to be seen as a hypocrite:

“It helped me be more of a leader. I didn’t realize that the younger kids were watching me, looking up to me. I don’t want to be a hypocrite. It helped me be more of a leader and acting that way. The adults tell you that the little kids watch you and copy you and you don’t think about it as much until they show you and tell you that they are”.

Others even acknowledged that being a peer mentor helped them determine their career path; for instance, Berenice, who felt like being a mentor allowed her to see herself as a future entrepreneur:

“Before I became a peer mentor I wanted to be a nurse. I then realized it’s not for me. I mean it kind of is but not all the way so right. I like taking charge us like some people like being a leader. So I decided to go in for business so I can open up a business in my community”.

Peer mentors in the study collectively agreed that being a peer mentor changed their perspective of potential careers. Kevin felt that being a peer mentor changed his life trajectory:

“Before being a peer mentor, I didn’t know what I wanted to do with my life. I didn’t really know if I wanted to go to college. And being a peer mentor and talking to all these kids and seeing how like the passion they always had … I found my passion for what I wanted to do. I can basically say talking to the kids (mentees) is what got to me where I’m at now and that is what I want to do forever”.

Another peer mentor, Tamara, discussed how she feels the work that she does with mentees is impactful on her own path:

“I am proud of what I have been able to do with my mentees. To even say I have a mentee, is such a huge thing. It makes me feel like I can make a difference and be an advisor for them. Change them in a way by me explaining everything that’s about to come as they go through high school. I’m kind of like, changing them in a way. I have always been a shy kid but I was like how am I supposed to present myself in front of these kids who might not take me seriously or anything. But then it kind of changed me in a way where I’m like wow, I’m not as shy anymore. That’s a big deal and I am learning to be proud of that”

Throughout the interviews, the peer mentors felt that the program improved their confidence and allowed for them to acknowledge their own good qualities that makes them outstanding leaders and mentors. The participants consistently discussed the pride that they felt in having a mentee to look up to them and believed the role of a peer mentor made them feel more important and valued.

### 4.2. Witnessing Their Strengths through Motivating Others

All peer mentors in the study mentioned in various iterations that being a peer mentor motivated them to become more intentional about being role models and improving how they viewed themselves. Patricia found this experience to be what motivates her to do well in school. Patricia admitted that she struggled with school before becoming a peer mentor and was worried about whether she would succeed as a peer mentor. However, Patricia attributes the role of being a peer mentor in encouraging her to focus more on doing well in school, herself, as a way to motivate others. As she mentioned:

“The program gave me a lot of motivation. You have to have motivation in yourself in order to push yourself. I normally do well in my job and school, but I tend to slack off a little bit. But now, how can I help these kids who are failing? I motivate myself to do better because I have other people looking up to me as a role model”.

Another peer mentor, Nicole, acknowledged that she knew she had a great reputation as being a peer mentor and wanted to maintain that by continuing to make good decisions for herself, so that her mentees would continue to look up to her:

“I feel like I am. I am not trying to be cocky, but I am because I built my reputation here at school. My guidance counselor tries to push me to work with kids because I am a kid myself and I know their struggle. I am learning to own that. I feel and know that I am a good mentor. I want them to keep looking up to me and know that if I can make it, they can make it too”.

The participants then highlighted their various strengths which they believe makes them good peer mentors. All the participants acknowledged that they all possessed different qualities but believed that their unique qualities are what helped them connect with their mentees. Jose acknowledged his sense of humor and ability to connect with his peer mentees through using laughter:

“I mean, of course I like to crack jokes, I have a sense of humor because not everything should be so dark and so serious all the times, some things should be a little more lighthearted. I still want to get my message across depending on like what I’m saying that specific time but I want to show them that you can be smart and funny. It’s okay to be both, life doesn’t have to always be so serious”.

Monica mentioned how she was never really the popular student until she became a peer mentor, and described her experience with the process as one that boosted her self-esteem.

“In a world where I sometimes don’t feel like everyone else thinks I’m good. My mentees and their friends think I’m awesome. So I feel awesome and I let everyone know I am. My mentees and other younger students that I don’t even mentor, feel comfortable coming up to me and like saying hi and stuff, take my advice. I help them with their homework, and I never saw myself doing that before being a peer mentor! It’s a great feeling and I didn’t know I had it in me”.

Peer mentors in the study also acknowledged the impact of being in a position where they had to support younger peers, made them more aware of their own insecurities and how to overcome them. One of the participants, Steven, mentioned that being a peer mentor instilled a level of confidence in him that allowed him to be more comfortable speaking in public as, previously, he had issues with stuttering and was generally considered to be shy and quiet:

“For me at least, being a peer mentor has actually opened up some doors, I was terrible when it came to speaking in public. I used to stutter, a lot … but now I’m not perfect of course, but I’m actually a lot better. I can speak, I can speak to you and not actually constantly stutter and keep a calm mind and a straight mind, but before, that’s not how it used to be. Especially the beginning of my peer mentoring, I was terrible. But because of the peer mentoring program I was able to get that experience, that knowledge to speak in public by knowing that I had to speak to my mentees a lot and get comfortable with them listening to me”.

Steven also mentioned that because he overcame his fears of speaking in public, he hopes that others can do the same: “I look at myself and would have never guessed that I would be here, speaking publicly with my stuttering issues. There are a lot of stutters out there, I hope they see me and think that if I can do it, they can do it too”. Peer mentors were very confident in their ability to not only lead, but to inspire the next generation. The participants collectively knew that their peers were depending on them, they all seemed to embody a sense of intrinsic motivation, and they acknowledged that the support of their guidance counselors and teachers who nurtured their strengths and self-worth allowed them to give back and lead as an example.

### 4.3. Family Influences

A majority of the peer mentors described how their own individual experiences with family influenced their role of being peer mentors and how they related to their mentees. One of the peer mentors interviewed, Kevin, whom earlier was introduced as being very confident and outspoken, mentioned that his father and his relationship was fragile due to his father leaving him and his mother when he was much younger, “ … my father, before he left me, he told me I’d never amount to, well I don’t want to say that word”. While Kevin mentioned that he did not have a Black male role model growing up, he knew he wanted to be a role model for his peers who may not have a Black male role model to look up to: “I want to be to my mentees what I never had”.

Patricia, another peer mentor, mentioned that because of her position in the family as the youngest child, she sees her role as a mentor as an opportunity to be a big sister for someone else:

“Being a peer mentor means a lot to me. I can be a big sister for once, which means a lot to me. I never got the chance to been seen in that way. I can relate to boy and girl issues. I can relate to them because I am a teenager, and that makes a difference”

In contrast, Tamara, who identifies as the oldest child in her immediate family, always wished she had an older sibling and is used to being a leader in her household. However, she feels that her position in her family as the oldest sibling helps her to be a better mentor:

“I feel like it’s helpful for them and for us peer mentors too because we realize were in that position sometimes. I’m the oldest so I would’ve liked being mentored by an older person. But I would’ve enjoyed being advised or told you know, you should be doing this or you should be doing this, this is what you’re going to see in high school. So mentoring 8th and 9th graders was helpful to me because it reminded me of what I do with my younger siblings and what I wished someone did for me when I was their age”.

Another peer mentor, Eric, shared how his family, including his mom, models what support looks like, and noted their relationship as influential on his role as a peer mentor:

“My support system is like my mom, [she] supports me through everything basically since I was alive I ain’t have no father until like a year or two maybe three, but she was always there. She was a consistent base and has always been there. Always telling me I can do this and that. Then I have my two dads, I have a stepdad and a real dad, all of them have taught me how to be there for others so I think that’s what makes me a good mentor and supporter”.

Collectively, peer mentors were very aware of how their personal lived experiences and family context impacted their ability to be effective peer mentors and their mentoring style with their mentees.

## 5. Discussion

Findings from this study provide insight into the impact of being a part of a peer mentoring program on a group of Black and Hispanic urban youth and suggest that the benefit of these programs is bidirectional. Specifically, participants’ awareness of their own leadership abilities, and how being a peer mentor shaped their view of self, was a main theme of the study. Although peer mentors are only a few years older than their peers, the role of being a peer mentor positively influenced their self-esteem, confidence, and leadership abilities. As suggested by our findings and consistent with the literature, peer mentorship programs can help high school students build meaningful connections and improve their self-worth through motivating others [40]. First, peer mentors were able to identify leadership abilities within themselves that most were not initially aware they possessed. James et al. (2014) conducted a study among peer mentors in the United Kingdom and found similar results; peer mentors developed the ability to handle a range of possible scenarios, and the experience of mentoring itself required the development of problem-solving skills [41]. It can be inferred that the experience of being a peer mentor supports youth to strengthen their leadership skills while also fostering positive development into adulthood. By giving youth at such a critical time of adolescence the ability to serve as a mentor and role model to their peers, programs such as these provide an opportunity for peer mentors to develop nurturing relationships and to become more mature in the process. According to youth empowerment research, leadership competency is a protective factor that is associated with positive developmental outcomes including improved self-esteem, confidence, higher educational attainment, and positive mental health outcomes among youth [42,43]. Secondly, peer mentors in the study identified their own individual strengths through their mentoring relationship which led to increased levels of self-worth and was a key motivating factor in continuing to inspire their peers. Consistent with research on the effects of peer mentoring from a mentors perspective, James et al. (2014) found in their study that youth who were involved in peer mentoring programs as mentors experienced personal growth, improved psychosocial wellbeing, and a sense of achievement. Peer mentoring programs can be incredibly valuable for both the mentor and mentee, providing opportunities for personal and social growth for both parties. Third, an interesting finding that emerged among the sample was the influence of family dynamics in their role as a peer mentor and their mentoring style. In addition to their interactions with their own siblings and family members, peer mentors in the study described how their own experiences of abandonment and trauma impacted how they related to their mentees. Douglas et al. [44] conducted a study with peer mentors who were affected by trauma and found that youth participants in their study tended to be motivated by their own lived experiences to motivate others through the act of being a peer mentor. While not all youth in the study described their experiences as being traumatic, peer mentors in the study collectively agreed that their experiences, their level of support from their families, and how they interacted with their siblings, all affected their ability and motivation to mentor. More research is needed on family dynamics and its specific role in the mentoring styles of young teen mentors.

## 6. Limitations

While this study has several strengths, limitations to this study are noted. First, although the sample size of 14 participants was sufficient for qualitative methodology [45], its generalizability is limited. Second, this study included mentors from one peer mentoring program, which was unstructured, and all mentors attended the same school; this may attribute to sampling bias. Although steps were taken to reduce coercion in recruitment—i.e., students were required to contact the researcher if they were interested in participating—this cannot be guaranteed as the researcher was known to the participants. Third, while all participants identified as either Black or Hispanic, the study team did not situate race or ethnicity in its research questions or findings to discover the impact of race/ethnicity on mentoring within the study. We encourage researchers to highlight the role of race and peer mentoring on youth of color in future studies. Lastly, this study did not interview mentees as the goal was to understand the benefits of being a peer mentor from the perspective of the mentor. While this study did not include interviews from their mentees, it is important to understand the confidence building effects that arose among peer mentors and the potential such an approach can have on a successful transition into adulthood. Despite these limitations, this study contributes significantly to the limited literature on peer mentoring programs.

## 7. Conclusions

Our findings provide insight on the benefits and development of peer mentoring from the peer mentor’s perspective. Based on this qualitative study, peer mentors revealed that being a mentor fostered their leadership abilities, they found value in motivating other peers, and acknowledged specific trends within their family context that shaped their mentoring style. Peer mentorship offers the ability for students to develop their leadership skills and acknowledges that a leader comes in many different forms. Peer mentorship allows a mentor to grow personally and professionally, while nurturing the growth of the mentee. Incorporating peer mentoring programs in high schools (especially those in underserved urban communities) can promote positive youth development in a school culture where students and teachers are lacking the resources in their community [24]. It is important to note that the youth who participated in this study all lived in an urban neighborhood, with a majority of them facing challenges that are common to urban youth such as disrupted family dynamics, exposure to violence, and lower educational attainment; creating a sustainable peer mentoring program can possibly serve as a means for urban communities to support positive development outcomes in urban youth [41]. While the burden of solving community issues should not lie on the youth alone, it is also important to note that highlighting the voices of high-achieving youth of color who attend an urban high school is not often seen in urban education research. Thus, it is essential that researchers continue to uplift their experiences as a way to learn from them and honor their strengths.

## Figures and Tables

**Table 1.** Participant Demographics.

Name (Alias)	Age	Race	Gender	Accepted into College (Yes or No)	College Bound	College Scholarship Offered
Monica	18	Black	Female	Yes	Yes	Yes
Anna	18	Hispanic	Female	Yes	Yes	Yes
Tamara	19	Hispanic	Female	Yes	Yes	Yes
Vicky	18	Hispanic	Female	Yes	Yes	Yes
Steven	18	Hispanic	Male	Yes	Undecided	Undecided
Jennifer	18	Hispanic	Female	Yes	Yes	Yes
Kevin	18	Black	Male	Yes	Yes	Yes
Shauna	18	Hispanic	Female	Yes	Yes	Yes
Crystal	18	Black	Female	Yes	Yes	Yes
Patrick	18	Hispanic	Male	Yes	Yes	Yes
Veronica	18	Hispanic	Female	Yes	Yes	Yes
Patricia	18	Hispanic	Female	Yes	Yes	Yes
George	18	Hispanic	Male	Yes	Yes	Yes
Eric	18	Black	Male	Yes	Yes	Yes

## Data Availability

The data is unavailable due to privacy restrictions.
